# Athlete Monitoring Systems in Elite Men's Basketball: Challenges, Recommendations, and Future Perspectives

**DOI:** 10.1155/2024/6326566

**Published:** 2024-10-18

**Authors:** Jakob Burger, Alexander-Stephan Henze, Thomas Voit, Richard Latzel, Othmar Moser

**Affiliations:** ^1^Division of Exercise Physiology and Metabolism, University of Bayreuth, Bayreuth, Germany; ^2^Sports and Rehabilitation Medicine, University Hospital Ulm, Ulm, Germany; ^3^Faculty of Applied Natural Sciences and Industrial Engineering, Deggendorf Institute of Technology, Deggendorf, Germany; ^4^Interdisciplinary Metabolic Medicine Research Group, Division of Endocrinology and Diabetology, Medical University of Graz, Graz, Austria

**Keywords:** athletic performance, fatigue, injury prevention, periodization, readiness, recovery, team sports, training load

## Abstract

Athlete monitoring systems (AMSs) provide a centralized platform for integrating, processing, analyzing, and graphing various monitoring data to help coaches manage the rigorous demands of elite men's basketball players, who frequently participate in high-stress games with minimal recovery time. This review synthesizes current challenges in deploying AMSs, underscores their role in injury prevention and performance optimization, and discusses technological advances that could enhance their utility. Key challenges include selecting appropriate monitoring methods based on human and financial resources, accuracy of data collection, real-time data processing, and personalization of training regimens. Due to the weaknesses and limitations of each monitoring method, it is recommended that both objective (e.g., external load data, heart rate measures, and biomarkers) and subjective (athlete-reported outcome measures) monitoring data be integrated into an AMS to provide a holistic insight of the athlete's health and readiness. In addition, decision support systems integrated into an AMS can help coaches quickly gain an overview of their players' current condition and make informed decisions about daily load and recovery management. In this context, future perspectives suggest the potential for AMSs to incorporate predictive analytics and artificial intelligence to further enhance decision-making processes in elite men's basketball. Our findings underscore the need for continued innovation and rigorous validation of AMS technologies to ensure they meet the evolving demands of professional sports environments.

## 1. Introduction

In elite team sports, constant demands on performance are systemically stressful but are required to perform at the highest potential level [1, 2]. For basketball, this is particularly intensified within the National Basketball Association (NBA), where the average number of games played per week during the regular season is 3.4. In addition, the competition calendar is tightly scheduled, especially for those who are playing on an international level [3]. This places high physical and mental demands, which is associated with an increased risk of injury, illness, nonfunctional overreaching, or overtraining [1, 2]. Therefore, targeted and individualized load management for training and competition as well as sensible planning of recovery periods are urgently needed [1, 2, 4, 5]. In practice, different technological solutions have been commercially available to support athletes in their general health and injury prevention.

Athlete monitoring systems (AMSs) are increasingly used to assist the entire supporting team (coaches, medics, physiotherapists etc.) [4, 6]. In addition to specific demands of the sports, sport- and environment-specific injuries and other sport-related health issues must be considered when planning and implementing AMS. In addition to injury monitoring as a cornerstone for injury prevention strategies, an AMS as a centralized software-based platform may include monitoring of athlete's external and internal loads, as well as their perceptual well-being and readiness for upcoming training sessions and competitions [7]. A variety of objective and subjective parameters are available for these purposes, especially since technological advances have brought new systems for tracking athletes, teams, and the ball indoors [4, 6, 8]. In a recent paper, Boullosa et al. pointed out that a specific “fine-tuning approach” with a combination of appropriate monitoring parameters should be pursued for different sports [9]. The initial consideration should be given to the sport-specific physical demands, which also varies by the position played (e.g., guard, forward, and center) within the team. The external load (the work performed by an athlete in a given time) is in basketball mainly characterized by repetitive, high-intensity movements such as sprints, accelerations and decelerations, changes of direction (CODs), jumps, and duels [10]. Due to the diverse nature of training regimens, the external load in training sessions is even more variable than those observed within regular match play [10]. The vast amount of tracking data generated must be processed, analyzed, and quickly presented in a simplistic manner to coaches, most of whom are not in detail statistically trained [11]. Software solutions, which often include decision-support systems such as color coding, may help to efficiently overcome this hurdle [11]. In addition, the resources available and the rules of the respective governing competitions must be considered when planning multimodal monitoring. For example, the tracking methods such as wearables used to quantify external load during training are not allowed during regular NBA matches, and within this vicious cycle, the optical tracking technology used there is not available to teams for practice [9]. This raises the question to which extent the two different tracking methods are comparable and whether they can be integrated into a concise AMS.

External load leads to an individual psychophysiological response at various functional levels, which is referred to as internal load and considered as the actual stimulus for adaptation [6]. For example, the percentage of maximum oxygen consumption (VO_2max_), lactate measurements, or training heart rate (HR_ex_) and training concepts derived from them can be used to assess internal load. In contrast, methods that assess parameters after training and competition do not, strictly speaking, evaluate the internal load itself, but the postexercise response to the internal load [6]. These include common perceptual measures such as the rating of perceived exertion (RPE), heart rate–based methods such as heart rate recovery (HRR), biomarkers from blood or saliva, and motor testing to assess neuromuscular status such as the countermovement jump (CMJ) [6]. However, these parameters can be used as surrogate parameters for the internal load of the functional level being assessed [6]. The internal load and the resulting fatigue are important contributors to an athlete's perceptual well-being, which is also influenced by other nonsport stressors, particularly at a mental and emotional level [12]. Multidimensional, sport-specific questionnaires represent the gold standard for assessing perceptual well-being [12, 13].

Following the “fine-tuning approach” of Boullosa et al., this narrative review aims to summarize the different methods of monitoring elite male basketball players and their limitations, to provide recommendations for the implementation of an AMS considering the challenges, and to highlight its future perspectives. Therefore, a search of the electronic database MEDLINE (“athlete monitoring” AND “basketball”) was performed via PubMed, yielding 451 hits. Articles were selected based on the expertise of two researchers (JB and ASH) and confirmed by the senior researcher (OM). In addition, literature used in systematic and nonsystematic reviews was used if the content laid within the scope of our review.

## 2. Athlete Monitoring in Elite Men's Basketball

### 2.1. Physical Demands

Basketball is characterized by a unique movement pattern and profile. This is evidenced by a very dynamic course of alternating offensive and defensive actions resulting in a complex requirement profile [14]. Short and highly intense actions such as CODs, accelerations, decelerations, (repeated) jumps, and side shuffles are commonly found movement patterns [14]. This results in the necessity of a well-developed aerobic capacity, which serves as the basis for the performance of repeated short and highly intense actions. Latzel et al. showed that the energetic profile of basketball might be up to 75% aerobic [15]. One single match of male basketball involves approximately 1000–3000 single actions, up to 150 sprints of 5–20 m in length and up to 70 jumps and landings are performed and in a match over 40 min, the athletes cover up to 8 km of distance [16–19].

In addition to the in-game activity, the accumulated load on the players is highest in nongame activities (e.g., training sessions) with a high interindividual variation depending on playing experience, playing status (starter, rotation player, or bench player), or position. This highlights the need for highly individualized monitoring of training and matches in basketball [10]. Established basketball-specific load profiles in sports science and practice primarily take speed, jump, COD, and intermittent endurance parameters into account [10]. Due to the demands of the sport, basketball training is multifaceted compromising different training types: court-based and basketball-specific regular team practice, position-specific small group or individual workouts, shooting practice, off-court resistance training, and conditioning [10].

### 2.2. Injury and Health Surveillance

**Figure 1:**
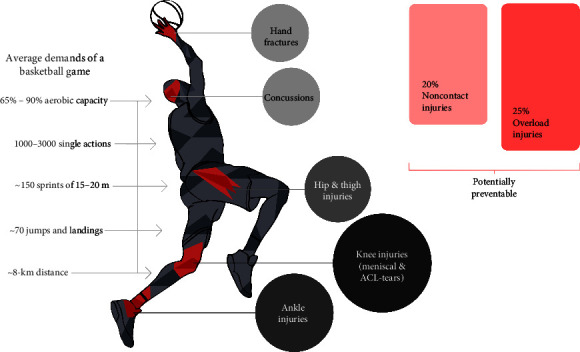
Average demands during a basketball game and the resulting most common basketball injuries including their prevention potential [15–18, 20–23].

Due to the physical demands described above, basketball is associated with an increased risk of injury that can affect various parts of the body [20]. A systematic review of epidemiological data on basketball injuries showed that the lower limb is the most commonly affected body region in basketball players regardless of age, gender, and performance level. In professional athletes, ankle (∼20%) and knee (∼18%) injuries were the most common [21]. A recent systematic review of injuries in the NBA revealed that the most common injuries were concussions, hand fractures, lower limb stress fractures, meniscal tears, and ACL tears [22]. A large-scale study examined injuries in the two top men's professional basketball leagues in Germany [23], defining an injury as any event during practice or competition that results in either medical costs or the player's inability to participate in future practices and/or games. It showed that approximately one-fifth of all injuries are noncontact injuries, which are considered potentially preventable. At least a quarter of all days lost to injury in German men's professional basketball is due to structural overload and could therefore be reduced by appropriate load and recovery management [23]. The lower limbs are particularly affected by acute injuries, accounting for ∼60% of all injuries [21, 23]. Each season, 70% of German professional male basketball players suffer at least one significant injury. The most commonly affected areas in this population are the ankles (∼18%), knees (∼14%), and thighs (∼10%) [23].  Figure 1 summarizes the physical demands of a basketball game and the resulting common acute injuries and their potential for prevention.

When reviewing epidemiological studies, it is important to note that no standardized definition of injury (or other health problems) is used, making comparisons challenging. It is also essential to highlight the types of cumulative measures (e.g., period prevalence, cumulative seasonal incidence, and average incidence over years) that are reported. For this reason, the International Olympic Committee (IOC) has adopted a consensus statement on the approach to epidemiological studies of injury and illness in sport [8]. The definitions and recommendations contained therein should be considered when implementing and conducting injury and health surveillance, which forms the basis of an AMS. A carefully structured system for monitoring injuries and other health problems in athletes should, first and foremost, assist the various stakeholders (e.g., coaches, trainers, medical staff, and sports management) in identifying the pertinent issues and potential risk factors within the specific environment [8]. It also serves as a benchmark for measuring the health effects of adjustments in load and recovery management, such as changes in training regimens (e.g., duration, density, volume, intensity, and frequency), the introduction of new prevention programs, or recovery strategies. It can also be used to evaluate the efficacy of newly implemented monitoring methods, which often require significant human and financial resources.

### 2.3. Monitoring of External and Internal Loads and Well-Being

Basketball practitioners need to evaluate differentially what external loads athletes were actually exposed to during training and competition. Time-motion analysis (TMA), a complex video-based method, has been used primarily to quantify physical demands in team sports [24]. However, this method is not suitable for daily monitoring [24]. Based on technological advances, more and more ways to track players have been developed in recent years: global navigation satellite systems (GNSS), including the global positioning system (GPS), local positioning systems (LPSs), and optical tracking [25].

#### 2.3.1. External Load

The easiest way of monitoring the external load of an activity might be exercise duration. It is used for the training volume and multiplied with an (internal) intensity measure such as heart rate or RPE to get a comparable training load (TL) [26]. Within this approach, methodological differences for quantification of session duration have been identified by Russell et al. [27]. There is no standard operating procedure (SOP) which content of the exercise session should be included or excluded, and there is a clear contrast between theory and practice. Consequently, the comparability of session and TL is questionable [27].

Apart from training duration, external load metrics commonly used in basketball are metrics of distance, measuring the cumulative distance with the possibility to measure the total and relative distance, and the distance in certain speed zones [14, 25]. To gather those numbers in basketball practice, LPS and inertial measurement units (IMUs) are used as a standard. The indoor application of GPS systems comes with significant inaccuracies and is therefore not recommended [28]. With technological advancements in recent years, optical tracking has become more relevant, providing real-time feedback without the necessity of wearables. Where regulations inhibit the usage of wearables during competition (e.g., NBA matches), optical tracking offers a promising perspective [25].

Measures of acceleration and deceleration, signified as the instantaneous maximum velocity rate (positive or negative), are commonly used in the quantification of external load [14, 25, 29]. They can be displayed as the number of momentum changes and the distance covered or time spent when accelerating or decelerating. Moreover, the immediate peak rate of position changes with metrics such as maximum speed, mean speed, or peak speed is taken into consideration [14, 25, 30].

Torres-Ronda et al. pointed out that there is a lack of consensus and standardization between systems and manufacturers in the application of thresholds and profiles for those metrics [25]. This makes it rather difficult to draw relevant comparisons between systems when thresholds or technological profiles are not matching. The careful selection of the specific metric profile, however, is vital for the effective application within the sport. Meaningful conclusions dependent on the context (e.g., injury prevention, performance enhancement, and rehabilitation) should be tailored individually [25].

Furthermore, in elite basketball, COD are an established measure used for the assessment of external load [25, 28]. Impacts on the players, referenced to gravity (*g*-value), are used alongside the metabolic power (power generated by an athlete relative to their mass) of the players [25, 31, 32].

As a kind of an umbrella metric, PlayerLoad (PL) comes as a key measure in basketball [25, 29]. It incorporates external load metrics, converting them into arbitrary units (AUs) over time, taking various acceleration-based parameters into account [28]. The relative measure of PL per minute (PL min^−1^) facilitates a comparison of external load between groups or individuals. Previous studies of Luteberget, Holme, and Spencer showed its validity in team sports settings, rendering it applicable for elite basketball [33, 34]. It is important to note that all PL-labeled metrics assess the same variables. Some systems only take two-dimensional (2D) data into account, neglecting acceleration in the *Z*-axis which limits their validity for basketball where quite some work is performed vertically [25].

A recent study of Ibáñez et al. examining external metrics in basketball showed positional differences, concluding that in basketball, not only the understanding of the varied intensity zones of external load is vital, but also recognizing the speed distribution within these zones as well as the behaviors influencing athletes' intensity levels are important [30]. This knowledge is essential for the design of effective training programs that ensure athletes' health and ability to perform [26, 27, 35, 36].

#### 2.3.2. Internal Load and Well-Being

Monitoring the actual internal load, i.e., recording internal load parameters such as heart rate or blood lactate during training or competition, comes with challenges in basketball. For this reason, surrogate parameters, which strictly speaking represent the response to an internal load, are commonly employed to assist coaches to better understand an athlete's response to training and competition, thereby enabling them to optimize their training [6]. Athlete self-reported measures (ASRMs), heart rate–based methods, and biomarkers are frequently used for this purpose [6, 37]. Like the internal load, the subsequent fatigue and recovery processes as well as the perceptual well-being (or wellness) depend on many factors and require individual consideration.

##### 2.3.2.1. Athlete-Reported Outcome Measures (AROMs)

A standard method that has been used for many years in the context of internal load monitoring is RPE, in which athletes rate their perceived exertion on a defined scale (e.g., the original Borg scale, category ratio [CR]-10 scale, or CR-100 scale) at the end of exercise [38]. This is also the basis of Foster's session RPE (sRPE) method, where sRPE is assessed within 30 min after the end of exercise using a modified Borg CR-10 scale [38] based on the question “How was your workout?” [39]. The sRPE is then multiplied by the individual training/competition duration to obtain the session load in AU [39]. The validity of the sRPE method in professional male basketball players was first investigated by Manzi et al. [40] The authors demonstrated a significant correlation between sRPE and heart rate responses and proposed this method as a single monitoring tool due to its noninvasive, easy-to-use, and cost-effective nature [37, 40]. Further studies of this method in semiprofessional male basketball players showed significant moderate relationships between sRPE and accelerometry-based measures of external load and indicated its superiority over heart rate–based methods for internal load monitoring in different training modes, especially for court-based training with multidirectional running drills [41, 42]. Svilar et al. showed a high correlation between RPE and sRPE and the total amount of accelerations, decelerations, and CODs in 300 training sessions of 13 professional basketball players [43]. Another observational study showed a high week-to-week-variation in total weekly TL assessed via sRPE in collegiate basketball players with higher weekly TLs in 1-game weeks and starting players [44]. In recent years, two independent observational studies have evaluated the sRPE method in professional male basketball players for each training session and match over the course of an entire season and showed lower total loads in regular weeks (one official match within a 7-day period) than in congested weeks (at least two official matches within a 7-day period) [45, 46]. Ferioli et al. observed higher weekly total loads and TLs in the last 6 weeks of the regular season than in the entire 6 weeks of the playoffs in ten professional male basketball players [47]. In summary, the sRPE method has been shown to be valid and reliable in a variety of sports and can be used regardless of gender, age, or performance level [48]. It has also been well studied in professional basketball in recent years and can be considered an easy-to-use part of an AMS. However, the use of sRPE must take into account the limitations inherent in subjective methods, such as the possibility of manipulation by the athlete. It should also be noted that, strictly speaking, sRPE is a hybrid method consisting of an external load parameter (session duration) and a surrogate for the internal load (RPE).

Another important domain of AROMs (or ASRMs) is the monitoring of perceptual well-being, which is influenced by training and competition load, team and individual performance, and nonsport stress and coping factors [12]. From a scientific point of view, multidimensional questionnaires are considered as the gold standard in athlete monitoring when it comes to measuring well-being [12, 13]. In practice, however, athletes and coaches prefer customized simple and short questionnaires [49, 50], which often lack validation studies [13]. The few studies that have examined the well-being of male professional basketball players in relation to other monitoring parameters [44–46] have predominantly used ratings based on Hooper and Mackinnon's recommendations [51]. While Hooper and Mackinnon's original recommendations included other categories (mood, enjoyment of training, irritability, and health), the term “Hooper questionnaire” is typically used in studies to describe the combination of the four categories “fatigue,” “stress,” “sleep,” and “muscle soreness” with the sum of each category rating being the “Hooper index” [45, 52]. A limited number of studies in professional basketball have employed validated questionnaires in monitoring. According to a recently published systematic review evaluating the relationship between AROMs and subsequent match performance in team sports [13], only one study with professional male basketball players and one study with semiprofessional basketball players met their inclusion criteria. The first used the profile of mood states (POMS) over a 5-week period [52] and the second used a modified 10-point total quality recovery (TQR) scale over an entire season [41].

In order to meet the needs of sports science and practice, the use of a short, sport-specific, multidimensional questionnaire such as the short recovery stress scale (SRSS) seems to be a suitable option for daily monitoring and should be further investigated in elite basketball. Finally, AROMs in the form of a simple Likert scale can be a time- and cost-effective solution for monitoring sleep quality [53] in addition to sleep duration, if they are not already included in the well-being questionnaire used.

##### 2.3.2.2. Heart Rate–Based Methods

Heart rate–based methods have been extensively used to objectively assess internal load in elite sports and basketball [54, 55] and to validate other load monitoring methods such as blood lactate [56] or AROMs [37, 40, 56–61]. Especially Banister's training impulse (TRIMP) [62] and Edwards' TL [63], also referred as the summated heart rate zone (SHRZ) model, have been widely used in team sports and also in basketball [40, 42, 64–66] and are considered as the gold standard by some researchers [40]. However, these models are based on observations from (running-based) endurance sports and their applicability to intermittent team sports remains limited. Banister's TRIMP is based on the average HR weighted by the relationship between HR and blood lactate as observed during an incremental test and the duration of the session [62]. In contrast, Edwards' TL or SHRZ model estimates TL by the time spent in five different zones in % of HR_max_ to which a linearly increasing coefficient is assigned (50%–60%: 1% to 90%–100%: 5) [63]. The study by Manzi et al. was the first to investigate these two HR-based models in professional basketball and showed that these models detected similar changes in weekly periodization patterns without separating different training modes [40]. Therefore, Clemente et al. evaluated eight semiprofessional male basketball players over 30 training sessions using the same methods and found that these HR-based models and the sRPE method estimated similar TLs when applied during repeated linear running, sprinting drills, and tactical/gameplay conditioning. In contrast, the models appear to have weaknesses in estimating the TL of more basketball-specific drills involving a large number of high-intensity, intermittent, and multidirectional movements [45]. These findings are consistent with those of Alexiou and Coutts, who concluded that HR-based methods are not well suited to quantify TLs at very high intensities or in competitive games [56]. The relatively wide HR ranges (10%) were identified as a weakness of the SHRZ model, so modified models with narrower HR ranges (5% and 2.5%) were also evaluated in basketball [67]. This study showed that the SHRZ 2.5 appears to be a better option when applied to basketball-specific training drills. In addition to the methodological shortcomings in terms of transferability to elite basketball, these HR-based methods also require additional resources for data collection and analysis. However, HR-based methods still offer a way to objectively assess the internal load in certain training modes, especially when modified models such as SHRZ 2.5 are used for calculation.

##### 2.3.2.3. Biomarkers

Biomarkers derived from blood, saliva, sweat, or urine promise objective measurements, which is why sports science and sports medicine professionals frequently utilize them to analyze an athlete's health and performance, fatigue, and recovery [48, 68]. However, biomarkers are subject to some circadian and ultradian variability independent of physical activity, have considerable interindividual variability, and often lack reference ranges in athletic populations [48, 69]. In addition, sample collection may be invasive (venous blood and capillary blood), and processing and instrument-based analysis are often time-consuming and expensive [69].

In professional men's basketball, biomarkers are primarily studied and used as surrogate markers of muscle stress (e.g., creatine kinase [CK] [54, 70–75], urea [74], and lactate dehydrogenase [LDH] [72–74]), metabolic stress (e.g., lactate [15, 54, 55, 70, 71, 73, 75–81] and urea [74]), chronic inflammation (e.g., C-reactive protein [CRP], cytokines [70, 74]), and brain function (e.g., S-100B [73]), or endocrine responses to acute and chronic stress (e.g., testosterone [T] [65, 72, 82–84] and cortisol [C] [65, 70, 72, 74, 82–85]). The majority of biomarker studies in professional male basketball players have been designed to investigate basketball-specific physical demands, the effects of training interventions or nutrient supplementation, and in the case of blood lactate, the validation of tests of submaximal endurance or anaerobic capacity. The few studies that have examined blood-based biomarkers in professional basketball players mainly focused on inflammatory and hormonal responses after official matches of the regular season [70, 74, 86]. An exception in terms of study duration is an observational study by Schelling et al. in which 20 professional basketball players underwent blood tests to assess hormonal state every 4–6 weeks for four consecutive seasons after a baseline examination [84]. In this study, a higher catabolic state (lower total plasma T:C ratio and higher C levels) was observed in all players in the last third of the regular season [84]. In addition, this study showed a dependence of hormonal responses on playing time and playing position [84]. However, the blood tests in this study were also performed in the context of competition (after a rest period of 24–36 h).

Given that monitoring procedures should impose the least possible additional burden on an athlete, capillary blood or even saliva is preferable [69]. Consequently, the latter is increasingly becoming the focus of elite basketball research, as evidenced by recent studies focusing mainly on metabolomics [87, 88] or salivary hormonal responses [65, 85, 86]. T, C, and their relationship to each other should provide insight into anabolic and catabolic processes and thus training and recovery effects [89]. Therefore, Kamaraukas et al. examined salivary T and C levels in different study populations (European level) on a weekly basis during a 5- or 6-week preseason period [65, 85, 86].

Regardless of the type of body fluid, Lee et al. identified some basic principles for the frequency and timing of biomarker testing to obtain the most valuable information about the athlete's state of health and recovery [48]. They included baseline measurements in the off-season to capture individual variations while the players were rested, defined measurements in a controlled environment during training and competition to capture fatigue-induced changes, and measurements at key points in the preparation period and competition phases [48]. Considering their recommendations, it is evident that calculating individualized reference ranges for the biomarkers that align with the physical demands of professional men's basketball is a logical next step. Therefore, in addition to *Z*-scores, a method published by Hecksteden et al. in 2017, which is based on a similar principle as the Athlete Biological Passport, can be considered [90]. Based on a sport-specific population, reference ranges for the recovered and fatigued states are calculated by progressively incorporating individual repeated measurements with a known recovery state in the sense of a two-point calibration [90]. This method has been demonstrated to be superior to a classification using group-based reference ranges in elite team sports for CK [91, 92], which is considered a key biomarker for monitoring elite team athletes [90].

### 2.4. AMSs: A Comprehensive Approach for Handling Players' Data

There are a variety of methods and parameters used to measure external load, internal load and resulting fatigue and recovery processes, and associated perceptual well-being. The resulting data must be processed, analyzed, and graphically displayed. To avoid the necessity of switching between different devices or software applications, a comprehensive AMS that provides all the necessary information to the responsible coaches seems ideal. As a decision support system (DSS), it should follow the IOC guidelines [8]. The system should enable the individual practitioner using the system to perform their role more effectively. Second, the system should provide clear and reliable information. The data collected must not be the data shown to the practitioner, but already be filtered and made accessible. Third, the system should outline a degree of goal achievement for the specific values set by the user [8]. In elite sports practice, AMS follows a similar pattern guiding the practitioner to the desired outputs. The process can be operationalized into three general steps: first, data collection (depending on external or internal measurements manual or semiautomatic input); second, data analysis; and third, data visualization with an interface that is accessible via an online dashboard. Various solutions on the market provide customized dashboards for coaches, athletes, performance departments, or technical support, depending on their role in or for the team.

A fundamental aspect of any AMS is injury and health surveillance. To facilitate comparisons of sustained injuries and illnesses, it is of utmost importance to use a standardized nomenclature of body regions and areas, as well as a concise terminology in general. The system can use unique identifiers for injuries (e.g., body map for the location of the injury), with the ability to clarify the injury specificity, note the expert's diagnosis along with the circumstances, and comprehensively track the time and sequence of injuries or other health problems. The severity is assessed by the loss of time due to the injury and illness and is also tracked by the system [8].

In addition to injury and health surveillance, an app-based entry of AROMs (sRPE, perceptual well-being, and sleep data) by the players is recommended. The system should offer an option to customize the timing of the questionnaire/data entry and provide a reminder to the athlete at a specified time (e.g., before or after practice and after awakening). The ability to customize the input is useful for individualized rehab programs or training groups that require different parameters.

The processing and analysis of the data should be more or less fully automated and visually presented to the user by a DSS that might include traffic light logic (e.g., red, yellow, and green) or other means of visualization to draw simple but meaningful conclusions [11]. The visualization should highlight certain noteworthy values (extreme values, outliers, and values deviating from the mean) and display them separately. To reach these conclusions and substantiate the findings, various forms of artificial intelligence (AI) have been employed, such as artificial neural networks, decision tree classifiers, and support vector machines [93].

For the responsible practitioner (e.g., head coach), the AMS should provide a clear indication and support his decision-making process (e.g., whether to rest a player).

## 3. Discussion

The use of an AMS with interfaces to all relevant data sources and an integrated decision support system appears to be a useful approach to assist those responsible for athletes, such as coaches and medical staff, in their daily work. From a scientific point of view, the efficacy of an AMS remains uncertain. Validation studies show that certain parameters correlate with athlete performance, but to the best of our knowledge, no study showed a significant reduction in injuries in elite youth and senior basketball. Furthermore, it is challenging to identify clear evidence to favor one metric over another. Some authors [94–96] point out that papers claiming to have found one single metric that can help to significantly reduce injuries are simply not methodologically sound and lack good scientific practice. A holistic, i.e., multivariate, monitoring system that is individualized not only to the needs of athletes but also to those of coaches seems to be a promising solution for elite basketball. However, this leads to complex (theoretical) models that try to describe the factors that influence injuries. The challenge lies in making complex theoretical models easily accessible to practitioners (coaches and athletes alike). Sports scientists and physicians need to keep in mind that the research they conduct can be very helpful in sports practice, but only if it can be applied by practitioners.

The inclusion of an excessive number of metrics in sports practice can potentially be overwhelming for the athlete, leading to a lack of compliance with data collection procedures [97]. This might be due to the disruption caused in the athletes' training routines. Although in a professional setting, clubs could force athletes' compliance, this could influence the data input. Effective and transparent communication could help create understanding and foster athletes' compliance. Continuous athlete monitoring can come with challenges such as the psychological burden caused by the ongoing questionnaires. Feedback toward the athletes on their data input appears to be an important factor for persistent collaboration in data collection. The support of the practitioners is crucial here in order to ensure the cooperation of athletes [98–100].

Each sports has its specific physical demands and unique movement profile, but the rather small effect sizes in a competitive sports context make it challenging to identify valid and stringent arguments. Hence, it seems logical to consider the findings of studies conducted in similar sports. Following up on the lack of standardization between different AMS technologies that have been mentioned in the literature before [25], we recommend increasing standardization by implementing more guidelines like the one handed out by the IOC on injury surveillance [8]. These guidelines should clearly set benchmarks like which Likert scale type to use, when to use which kind of questionnaire, or how to handle the generated data correctly.

Finch's translating research into injury prevention practice (TRIPP) framework, Bishop's applied research model for the sports sciences (ARMSS), and others call for the transfer and adoption of findings from science into sports practice. The assertions that enhanced medical approaches are not appropriate for sports practice and that study designs are too complex will prevent progress in applied research [101, 102]. The objective is therefore to bridge this gap to facilitate the acceptance from theoretical findings into elite sports practice [103]. This represents one of the most important tasks for applied sports scientists today, as it is a key factor in creating real-world progress and effectively reducing injury incidence. For practitioners, the effort involved to get insights, e.g., the simplicity of interfaces or having one platform that integrates all metrics in practice are critical points to address when it comes to creating an impact. The expert who collects the data must have rather seamless integration of data and everything stored in one place, and the athlete or coach must get useful feedback on their data input on the other hand. In the future, even more impact can be generated through the usage of AI enhancing the effectiveness to generate meaningful impact. With the vast amount of data being collected, ethical considerations alongside data security and privacy issues come to mind since the generated insights are highly sensitive. Technically, the data belong to the athletes' and are only made accessible through contract regulations and compliance of the athletes with the procedure.

In the future, the research on AMSs in elite team sports and especially in basketball should focus on the efficacy and more precisely on how exactly they help in reducing outage times, therefore, to foster successful careers and increasing athlete and team performance. Technological advancements such as AI can help but must be carefully thought of instead of blind integration, especially when thinking of ethical or data security issues. In practice, the communication of all stakeholders comes as the key factor for successful system integration and athlete compliance.

## Data Availability

Data sharing is not applicable to this article as no datasets were generated or analyzed during the current study.
